# Botanical Origin, Phytochemical Profile, and Antioxidant Activity of Bee Pollen from the Mila Region, Algeria

**DOI:** 10.3390/antiox14030291

**Published:** 2025-02-28

**Authors:** Nassiba Boulfous, Hakima Belattar, Roberto Ambra, Gianni Pastore, Asma Ghorab

**Affiliations:** 1 Laboratory of Natural Sciences and Materials, Institute of Natural Sciences and Life, University Center Abdelhafid Boussouf, Mila 43000, Algeria; h.belattar@centre-univ-mila.dz; 2 CREA (Council for Agricultural Research and Economics), Research Centre for Food and Nutrition, 00178 Rome, Italy; roberto.ambra@crea.gov.it (R.A.); giovanni.pastore@crea.gov.it (G.P.); 3 Department of Vegetal Biology and Soil Sciences, Facultade de Ciencias, Universidade de Vigo, 32004 Ourense, Spain; asma.ghorab@uvigo.gal

**Keywords:** bee pollen, botanical origin, phenolic contents, antioxidant activity, HPLC, Algeria

## Abstract

Bee pollen is a complex mixture of floral pollen, and nectar fused substances from bee saliva. It is well known for its high content of proteins, carbohydrates, lipids, vitamins, and phenolic compounds, among various other physiologically active components. Its composition varies significantly depending on its botanical sources and environmental conditions. This study investigates the relationship between the botanical origins, chemical compositions, and antioxidant activities of 15 bee pollen samples collected from different areas in the Mila region of northeastern Algeria. The botanical origins were identified using a palynological method, categorizing 11 samples as monofloral and the rest as polyfloral. The total phenolic and flavonoid contents were measured, and their antioxidant capacities were evaluated through DPPH radical scavenging assay, reducing power assay (FRAP), and total antioxidant capacity (TAC). HPLC analysis was conducted to measure 17 phenolic compounds. The data indicated that the total phenolic content (TPC) and flavonoid content (TFC) ranged from 7.72 ± 0.29 to 23.49 ± 1.48 mg GAE/g and from 1.48 ± 0.00 to 5.57 ± 0.27 mg QE/g of pollen, respectively. The variations in the concentration of bioactive compounds among samples led to significant differences in their antioxidant activities: DPPH (IC_50_: 1.12 ± 0.15 to 0.21 ± 0.00 mg/mL), FRAP (EC_50_: 0.06 ± 0.00 to 0.29 ± 0.00 mg/mL), and TAC (262.17 ± 3.41 to 677.14 ± 12.81 EAA mg/100 g of bee pollen), with the most active samples being monofloral types from *Cistus* type and *Brassica* type. A strong correlation was observed between TPC, TFC, and antioxidant activity. Among the 17 tested compounds, only coumaric acid, rutin, myricetin, naringenin, resveratrol, and kaempferol were detected. In conclusion, both monofloral and polyfloral bee pollen samples represent a rich source of polyphenols with significant antioxidant potential.

## 1. Introduction

Bee pollen, a widely recognized honeybee product, is valued for its biologically active compounds and use as a natural remedy. It results from bees collecting pollen from flowers and mixing it with nectar and their secretions [1]. Typically, bee pollen contains carbohydrates (40–85%), proteins (14–30%), and lipids (1–10%), along with vitamins, minerals, carotenoids, phenolics, and trace elements. Over 250 bioactive compounds have been identified in bee pollen [2].

In apitherapy, it is used for its medicinal properties, including antimicrobial, anti-inflammatory, antiviral, analgesic, immunostimulant, hepatoprotective, and antioxidant effects [2]. Studies emphasize its strong antioxidant properties, which contribute to cellular protection and overall health by reducing oxidative stress [3,4,5,6,7].

The quality and antioxidant power of bee pollen is influenced by factors such as the geographical location where it is collected (and the specific plants bees forage on), the time of year, weather conditions, and how the pollen is processed. Determining its botanical source is essential, and this is primarily performed through palynology, which involves microscopic analysis of pollen grains to trace their plant origins [8]. Flavonoids like isoquercetin, myricetin, tricetin, quercetin, luteolin, selagin, kaempferol, and isorhamnetin have been identified in bee pollen samples from Brazil [9].

Algeria, located in North Africa, has a Mediterranean climate and boasts a rich plant diversity with 4000 species, including 2500 in its northern region, 10% of which are endemic. Examples include *Erysimum cheiri* (Brassicaceae) and *Hypochaeris saldensis* (Asteraceae). Numerous plants, such as eucalyptus, orange trees, sunflowers, clover, French honeysuckle, rosemary, and thyme, as well as natural forests like pine, provide nectar and pollen for bees, enabling year-round honey production. The region’s diverse flora and government support have fostered the growth of beekeeping [10,11]. Honey and propolis have been extensively studied by researchers [12,13,14,15,16,17,18,19,20]. On the other hand, only a few studies have characterized the antioxidant properties of Algerian bee pollens.

In this context, the current work is devoted to determining the botanical origins, analyzing the antioxidant capacity and polyphenolic composition using HPLC of bee pollen collected from the Mila region of northeastern Algeria. By examining these aspects, the study seeks to understand the relationships between the botanical sources of the pollen, its phenolic content, and its antioxidant properties.

## 2. Materials and Methods

### 2.1. Collection of Samples

**Figure 1:**
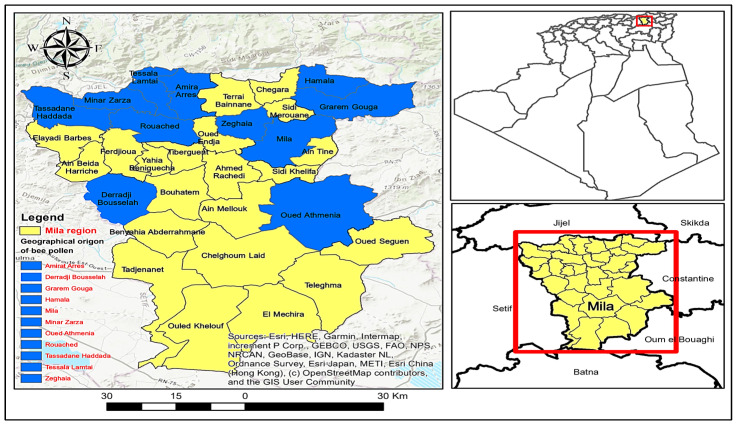
Geographic distribution origin by municipality (in blue) of the bee pollen samples collected the Mila region (northeastern Algeria). Created with ArcGIS 10.8.

In this study, 15 bee pollen samples from the Mila region (northeastern Algeria) were analyzed. Bee pollen samples were collected by local beekeepers using pollen traps from *Apis mellifera* between 2022 and 2023 years from different locations, and stored in the freezer at 4 °C until further analysis (Table 1, Figure 1).

### 2.2. Determination of Botanical Origin

Botanical origin was determined using a melissopalynological procedure [21]. First, the original samples were homogenized, then 1 g was weighed and placed in separate vials. Subsequently, a colorimetric separation using a white light and a black background was carried out to obtain different subsamples, which were weighted and dissolved in distilled water (15 mL). The solutions were shaken for 10 min and at 4500 rpm for 5 min centrifuged. An aliquot of 100 μL was taken from the sediment to prepare the slides. An optical microscope was used to identify the botanical origin of the different subsamples. The pollen spectra of the samples were determined considering the weight of each subsample and its botanical origin. The results were expressed in percentages.

Bee pollen samples were categorized microscopically into four sections based on the abundance of pollen grains: dominant pollen (≥45%), secondary pollen (16–45%), significant minor pollen (>3–15%), and minor pollen (<3%). The dominant pollen indicates the botanical origin of the bee pollen. However, identifying pollen grains from the same genus or family can be challenging. In such cases, only the genus or family name is specified, such as *Anthemis* sp., *Taraxacum* sp., or Asteraceae. According to Louveaux et al. [21], a sample is classified as monofloral if the percentage of a specific pollen type exceeds 45% of the total.

### 2.3. Preparation of Bee Pollen Extracts

Ethanolic extracts (EEPs) of the bee pollen samples were prepared according to the method of Almaraz-Abarca et al. [22], with minor modifications. The EEP was prepared by mixing 10 g of crushed bee pollen, with a precision of 0.01 g in 100 mL final volume 80% (*v*/*v*) aqueous ethanol for 24 h at room temperature. The sample was filtered under reduced atmosphere pressure using a water pump. The filtrate obtained was centrifuged at 10,000 rpm for 10 min, and then it was evaporated under reduced pressure in a rotary vacuum evaporator. The evaporated extract was dried in a laboratory incubator at 38 °C until a solid mass was obtained, and stored in the freezer at 4 °C until further analysis.

### 2.4. Phytochemical Analysis of Samples

#### 2.4.1. Assessment of Total Phenol Content

The determination of the total phenolic content (TPC) was carried out based on the method developed by Singleton and Rossi [23], adapted to bee pollen. Briefly, 0.2 mL of the extract (2 mg/mL) was mixed with Folin–Ciocalteu’s reagent (1 mL). After 3 min, 0.8 mL of 7.5% sodium carbonate was added to the mixture. The solution was kept in the dark at room temperature for 2 h, and the absorbance was measured at 765 nm. The total phenolic content was expressed as mg gallic acid equivalents (GAE) per g bee pollen. All measurements were performed in triplicate.

#### 2.4.2. Assessment of Total Flavonoid Content

The total flavonoid content (TFC) was determined using a modified version of the method described by Arvouet-Grand et al. [24]. Specifically, 0.75 mL of bee pollen extract (2 mg/mL) was mixed with 0.75 mL of a 2% aluminum chloride solution. This mixture was then incubated for 15 min at room temperature. Subsequently, the absorbance of the solution was measured at 430 nm. A calibration curve was generated using quercetin as a standard, and the flavonoid content of the bee pollen extract was determined by applying the linear regression equation of the calibration curve. The results were expressed as milligrams of quercetin equivalents (QE) per gram of bee pollen. All measurements were performed in triplicate.

#### 2.4.3. DPPH Radical Scavenging Assay

The ability of bee pollen extracts to scavenge free radicals was evaluated using the DPPH method, which measures the antioxidant’s capacity to neutralize the stable 2,2-diphenyl-1-picrylhydrazyl radical using a modified method described by Tomás et al. [25]. Specifically, 0.75 mL of phenolic extracts, with concentrations ranging from 3 to 0.43 mg/mL, were mixed with 0.75 mL of DPPH solution (0.04 mg/mL). The absorbance of the resulting mixture was measured at 515 nm. The percentage of radical inhibition was calculated using the following equation:

% Inhibition = [(A_DPPH_ − A_Sample_)/A_DPPH_] × 100

The EC_50_ value, representing the concentration of the extract required to inhibit 50% of DPPH radicals, was determined by plotting the percentage of radical inhibition against the extract concentration.

#### 2.4.4. Reducing Power (FRAP)

The FRAP method, which relies on the reduction in the Fe^3^⁺–TPTZ (ferric tripyridyltriazine) complex to its ferrous form under acidic conditions, was conducted following a previously described method [26]. A 0.25 mL aliquot of bee pollen extract (with concentrations ranging from 3 to 0.43 mg/mL) was combined with 1.25 mL of sodium phosphate buffer (pH 6.6). To this, 1.25 mL of 1% potassium ferricyanide was added, and the mixture was incubated at 50 °C for 20 min. Next, 1.25 mL of 10% trichloroacetic acid was added. The solution was then centrifuged at 3000× *g* for 10 min, and 1.25 mL of the supernatant was transferred to a new tube. Afterward, 1.25 mL of water and 0.25 mL of 0.1% ferric chloride were added, and the absorbance was measured at 700 nm. Extract concentration providing 0.5 of absorbance (EC_50_) was calculated from the graph of absorbance against extract concentration in the solution. Ascorbic acid was used as positive control. Tests were conducted in triplicate, and the results were given as mean ± SD.

#### 2.4.5. Total Antioxidant Capacity

The total antioxidant capacity (TAC) was measured using the ammonium molybdate colorimetric method described by Prieto et al. [27]. This method is based on the reduction of molybdenum (VI) to molybdenum (V) by antioxidants present in the extracts, leading to the formation of green phosphate/Mo (V) complexes, which can be quantitatively measured. In brief, 0.1 mL of the ethanolic bee pollen extract was combined with 1 mL of reagent solution (0.6 M sulfuric acid, 28 mM sodium phosphate, and 4 mM ammonium molybdate). The mixture was sealed and incubated in a thermal block at 95 °C for 90 min. The absorbance of the reaction mixture was then measured at 695 nm, using a blank for reference. Ascorbic acid was used as the standard for calibration, and the results were expressed as milligrams of ascorbic acid equivalent per 100 g of bee pollen.

### 2.5. Determination of Phenolic Compounds by HPLC

The analysis of phenolic acid compounds in ethanolic pollen extracts was performed using a Thermo Scientific Vanquish HPLC system, equipped with a Diode Array Detector (DAD) set to 280, 300, 320, and 350 nm. Spectra were continuously recorded in the range of 210–600 nm, with a data collection rate of 10.0 Hz. The system also included a Vanquish Autosampler maintained at 6 °C. A 250 × 4.6 mm C18 Luna column (Phenomenex, Torrance, CA, USA) was used, kept at 25 °C. The mobile phase consisted of 0.5% formic acid in water (phase A) and acetonitrile (phase B). The gradient used for analysis was as follows: 95% A for 1 min; 90% A for 4 min; 90% A for 5 min; 85% A for 47 min; 75% A for 6 min; 48% A for 15 min, followed by 13 min of re-equilibration with 95% A between runs. Standards were prepared at approximately 1 mg/mL, then diluted to create calibration curves (ranging from 0 to 50 µg/mL) for various phenolic acids, including gallic acid, trans-cinnamic acid, caffeic acid, syringic acid, ferulic acid, p-coumaric acid, hydroxycinnamic acid, vanillic acid, chlorogenic acid, kaempferol, epicatechin, naringenin, resveratrol, quercetin, myricetin, rutin, and catechin. The standards used for HPLC analysis were obtained from Sigma-Aldrich (Merk Life Science S.r.l., Milan, Italy). The limits of detection (LOD) and quantification (LOQ) values are provided in Table 2. The sample was injected at room temperature with a volume of 20 μL.

### 2.6. Statistical Analysis

All experiments were conducted in triplicate, and the results were expressed as mean ± standard deviation (SD). The data were processed using R-4.4.2.2 (2024). A one-way analysis of variance (ANOVA), followed by Tukey’s multiple comparison test, was performed to determine statistical significance, with *p*-values less than 0.05 considered significant. Furthermore, Pearson’s correlation coefficients were calculated to evaluate the relationships between the analyzed parameters.

## 3. Results

### 3.1. Botanical Originof Bee Pollen

**Figure 2:**
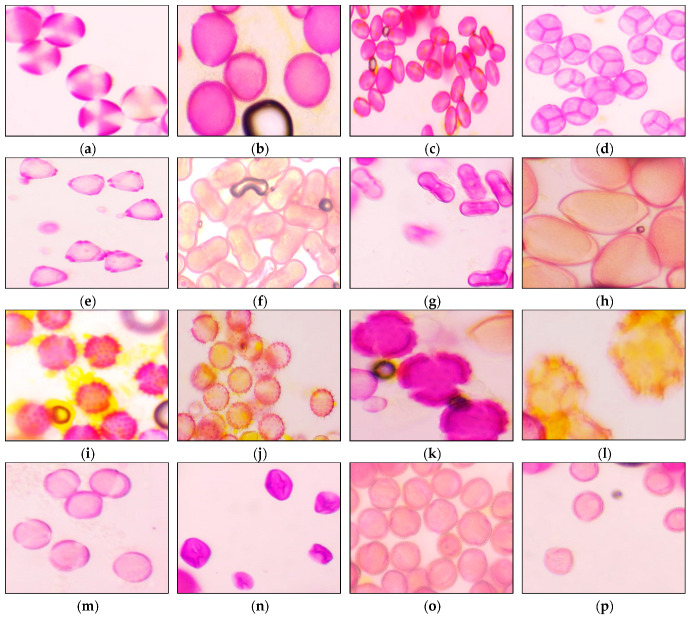
Microscopic appearance of the different pollen types found in Algerian pollen samples. (**a**) *Brassica* type. (**b**) *Cistus* type. (**c**) *Hedysarum coronarium*. (**d**) *Erica* sp. (**e**) *Eucalyptus* sp. (**f**) Apiaceae. (**g**) *Pimpinella anisum*. (**h**) Liliaceae. (**i**) *Aster* type. (**j**) *Helianthus annuus*. (**k**) *Carduus* type. (**l**) *Taraxacum* type. (**m**) *Tamarix* sp. (**n**) *Rubus* sp. (**o**) *Quercus* sp. (**p**) *Olea europaea*.

The results of the palynological analysis of bee pollen samples are summarized in Table 3 and Figure 2.

Among the analyzed samples, 30 pollen types from 17 families were identified. The most diverse families were Asteraceae, with 7 types, and Fabaceae, with 6 types. The remaining families were represented by only one or two types. The most frequently occurring pollen types were *Cistus*, *Brassica*, and various Asteraceae, appearing in nearly all samples.

The analysis classified eleven samples as monofloral and four samples as polyfloral. Samples P5, P8, P14, and P15 exhibited pollen from various botanical origins at different relative frequencies, as shown in Table 3. The monofloral samples were identified as originating from the following types: *Cistus* (P1, P2, P3, and P11), *Brassica* (P4, P6, P7, and P12), *Pimpinella anisum* (P9), *Erica* sp., (P10), and *Aster* (P13).

### 3.2. Concentration of Total Phenols, Flavonoids, and Antioxidant Capacity of Bee Pollen Extracts

Table 4 provides a summary of the total phenolic content, flavonoid levels, and antioxidant capacity of bee pollen.

The TPC of the tested samples ranged from 7.72 ± 0.29 to 23.49 ± 1.48 mg GAE/g. The highest TPC was recorded in sample P2, followed by P3 and P4, with values of 19.69 ± 0.69 GAE/g and 19.05 ± 1.90 GAE/g, respectively. The lowest TPC was observed in sample P13.

The TFC ranged from 1.48 ± 0.00 to 5.57 ± 0.27 mg QE/g of pollen. Sample P9 had the highest flavonoid content (5.57 ± 0.27 mg QE/g of pollen), followed closely by samples P14, P5, and P10, with values of 4.98 ± 0.09, 4.74 ± 0.24, and 4.28 ± 0.31 mg QE/g, respectively. The lowest TFC was found in sample P13, accordingly with the lowest. Interestingly, a contrasting trend was observed in samples P2, P3, and P4, where higher TPC was accompanied by lower TFC.

Different assays were employed to evaluate the antioxidant potential of bee pollen extracts, including the DPPH free radical scavenging activity, the reducing power assay (FRAP), and the molybdate ion reduction assay. The results are shown in Table 4.

DPPH radical scavenging activities ranged from IC_50_ 1.12 ± 0.15 mg/mL to IC_50_ 0.21 ± 0.00 mg/mL. The lowest IC_50_ value indicates high radical scavenging activity. The monofloral samples (*Brassica* type) P12 and P4 exhibited the highest antioxidant activity (0.21 mg/mL, 0.25 mg/mL, respectively) together with *Cistus* type monofloral bee pollen (P1), followed by P2 (0.34 ± 0.04 mg/mL), P8 (0.38 ± 0.01 mg/mL), P6 (0.42 ± 0.00 mg/mL), and P7 (0.50 ± 0.04 mg/mL).

The reducing power method revealed that sample P3 demonstrated the highest inhibition (EC_50_ 0.06 ± 0.00 mg/mL), while P11 showed the lowest inhibition (EC_50_ 0.29 ± 0.00 mg/mL). The antioxidant capacity of the samples decreased in the following order: P13 > P15 > P14 > P6, P8, P10 > P1, P7, P9 > P5 > P4, P2 > P12.

The total antioxidant capacity (TAC) values ranged from 262.17 ± 3.41 to 677.14 ± 12.81 EAA mg/100 g. Sample P12 exhibited the highest antioxidant activity (677.14 ± 12.81 EAA mg/100 g), followed by P5 (672.36 ± 3.41 EAA mg/100 g), P4 (612.54 ± 5.12 EAA mg/100 g), and P7 (595.96 ± 6.83 EAA mg/100 g). Conversely, P6 and P2, representing monofloral *Brassica* and *Cistus* types, respectively, showed the lowest activity. Significant differences were found between the mean values for all the variables analyzed in the bee pollen samples (*p* < 0.05).

### 3.3. Phenolic Compounds by HPLC Analysis

**Figure 3:**
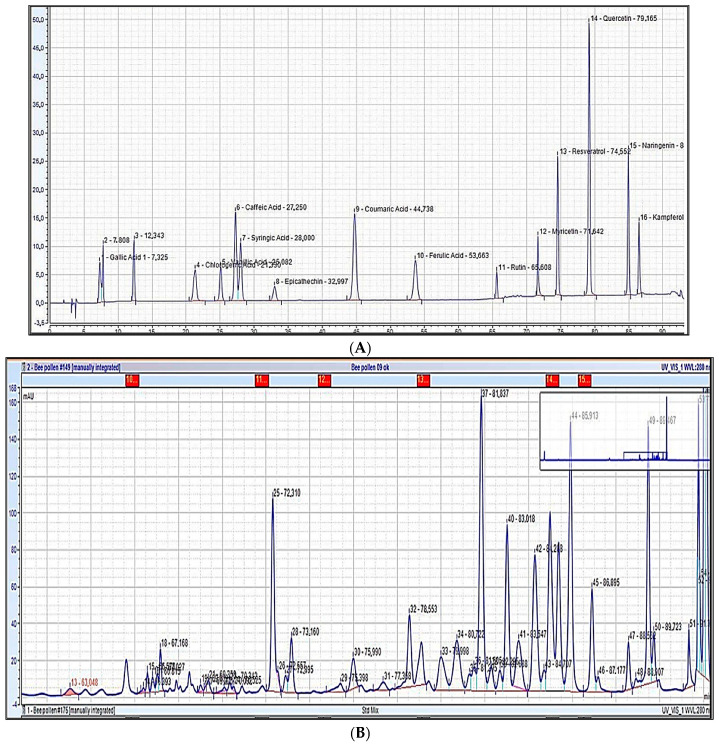
Chromatogram of the standards (**A**). As an example, the chromatogram of sample 9, zoomed with RT > 40 min (**B**).

In this study, phenolic compounds in ethanolic bee pollen extracts were analyzed using Thermo Scientific Vanquish HPLC. Figure 3A,B, illustrate chromatograms for phenolic standards and the phenolic profile of bee pollen extracts, respectively, using sample P9 as an example. Table 5 outlines the phenolic compounds (µg/g) identified in the extracts.

Out of 17 tested molecules, only p-coumaric acid, rutin, myricetin, naringenin, resveratrol and kaempferol were detected. Notably, high levels of rutin (11.4–1052.7 µg/g), myricetin (10.5–1418.1 µg/g), and naringenin (7.6–1418.1 µg/g) were found. p-coumaric Acid was absent in samples P1, P2, P3, P7, P11, P14, and P15. Sample P9 had the highest amounts of rutin (1052.7 µg/g) and naringenin (1418.1 µg/g) and the second-highest level of myricetin (1062.1 µg/g), correlating with its high flavonoid content (5.57 ± 0.27 mg/100 g).

### 3.4. Correlation Among Phenolic Compounds and Antioxidant Activity

**Figure 4:**
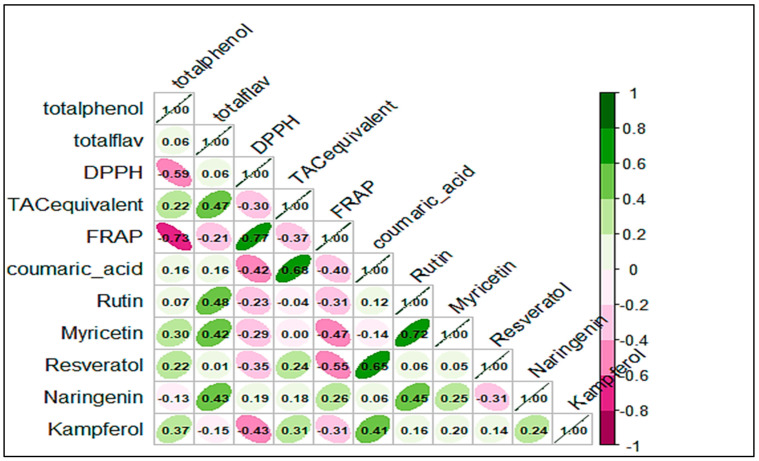
Pearson Correlation shown by the parameters studied in bee pollen samples from different botanical origins.

Using Pearson’s correlation (Figure 4), this study identified a strong positive correlation between total phenolic content (TPC) and kaempferol (r = 0.368), TPC and myricetin (r = 0.298), total flavonoid content (TFC), and total antioxidant capacity (TAC) (r = 0.472), TFC and rutin (r = 0.482), TFC and myricetin (r = 0.417), TFC and naringenin (r = 0.428). These correlations are explained by the fact that rutin, myricetin, and naringenin belong to the flavonoid family. Additionally, DPPH radical scavenging activity correlated positively with FRAP. A positive correlation was also observed between TAC and specific phenolic compounds, such as coumaric acid and kaempferol. There was further evidence of relationships between coumaric acid with resveratrol and kaempferol, as well as between rutin with myricetin and naringenin. However, a negative correlation was observed between TPC and both DPPH and FRAP (r = −0.594 and r = −0.734, respectively), which was attributed to the EC_50_ expression of these assays, where lower values indicate higher activity. Negative correlations were also noted between DPPH and TAC, resveratrol, and kaempferol, as well as between FRAP and TAC, coumaric acid, myricetin, and resveratrol. Among phenolic compounds, only naringenin and resveratrol showed a weak negative correlation. Pollen sample P12, belonging to the *Brassica* type with average TPC and TFC levels, showed the highest activity in both DPPH and TAC assays and ranked second in FRAP. Conversely, monofloral bee pollen P13 (*Aster* type) with low TPC and TFC exhibited the lowest FRAP activity.

## 4. Discussion

Bee pollen is a highly nutritious natural product, rich in antioxidants, carbohydrates, proteins, and essential amino. It also contains unsaturated fatty acids, vitamins, and minerals. Due to its nutritional and bioactive properties, bee pollen is widely used in dietary supplements, functional foods, and nutraceuticals for its health-promoting benefits. Its bioactive compounds, especially phenolic acids and flavonoids, contribute to their biological activities. Since pollen is derived from various plant species, its quality, safety, and characterization are determined by its botanical and geographical origins [28,29,30].

The botanical analysis of the bee pollen samples from this research highlights the rich floral diversity of the Mila region (northeastern Algeria), influenced by its Mediterranean climate, orography, and human activity. Most identified pollen types originate from spontaneous species with high melliferous potential, supporting the sustainable growth of beekeeping in the region [10,16,31]. With the exception of the Brassicaceae family, all the mentioned families are recognized as excellent pollen sources. However, most species within the Brassicaceae and Asteraceae families are primarily classified as nectar-producing. The Fabaceae family is known for its strong nectar and pollen production potential [32]. The dominant pollen types in the samples were *Cistus* and *Brassica*, suggesting that honeybees in the Mila region primarily collect these types for sustenance. Almost all samples were monofloral (11 samples). Carpes et al. [33] highlighted that the nutritional quality of certain pollen types makes bees favor a single floral source. Similarly, Aličić et al. [34] noted that bees typically visit the same plant species when collecting pollen, resulting in predominantly monofloral pollen with minor contributions from other species. There is limited data on the characterization of Algerian pollen in the literature. Bakchiche et al. [35] investigated pollen from the Laghouat region and identified taxa from families such as Asteraceae, Betulaceae, Boraginaceae, Brassicaceae, Cistaceae, Ericaceae, Fabaceae, Liliaceae, Myrtaceae, Salicaceae, and Rosaceae, with Brassicaceae being dominant. Mokhtari et al. [11] analyzed eight bee pollen samples from different Algerian regions and classified them as polyfloral, identifying 40 pollen types across 22 botanical families. The most diverse families included Asteraceae (8 types), Brassicaceae (4 types), and Cistaceae, Fabaceae, and Fagaceae (3 types each). These pollen types were also noted in other Algerian bee products, especially honey [16,20,36,37]. Similarly to our findings, El Ghouizi et al. [38] attributed the botanical origin of eight monofloral bee pollen samples to the Apiaceae, Fabaceae, Resedaceae, Rosaceae, and Lamiaceae families. Significant variation in pollen composition was observed, likely due to the diversity of environmental flora at different altitudes where beehives are located. Furthermore, bee pollen samples collected from the same or nearby areas can reflect different plant sources. This variation may depend on the dominant plant species in the vicinity of the hives and the foraging preferences of honeybees [26].

Several studies across various countries have investigated the total bioactive components of bee pollen. The TPC and TFC of Algerian bee pollen were similar to those found in samples from Marocco by El Ghouizi et al. [38], who reported TPC in monofloral bee pollen ranging between 8.070 ± 1.037 mg GAE/g and 32.387 ± 0.148 mg GAE/g, and TFC between 0.202 ± 0.044 mg QE/g and 6.30 ± 0.37 mg QE/g. The TPC values in our samples are consistent also with the results reported by Ilie et al. [39] for Romanian bee pollen. However, our TFC values are notably higher. In addition, we obtained higher levels of TPC and TFC when compared with the findings of Aylanc et al. [26]. A recent study in Algeria by Hemmami et al. [40] showed TPC values ranging from 3.79 to 9.17 mg GAE/g and TFC values between 2.07 and 5.50 mg QE/g. Compared to other studies involving bee pollen samples from Algeria, Brazil, Northwest Spain, Turkey, and India [5,7,11,18,28,41], our extracts exhibited lower amounts of total phenols and flavonoids. A high phenolic content does not necessarily correspond to elevated flavonoid levels, a finding consistent with previous research [26]. This discrepancy was observed in certain samples.

Bee pollen exhibits strong antioxidant potential due to its rich polyphenolic content. Various methods are used to assess its antioxidant activity, as different compounds require specific treatments and yield varying responses [29]. According to the literature, our DPPH radical scavenging assay results are consistent with those reported for Brazilian bee pollen, where the highest antioxidant activity was observed in the ethanolic extract of *Brassica napus* pollen [42]. However, our findings exceed the previously documented range of 7.77 to 1.68 mg/mL [6]. In addition, the methanolic extract of coriander bee pollen from India was revealed as a potent free radicals inhibitor (93.75 ± 0.05%) due to a minimum IC_50_ value (0.006 ± 0.21 mg/mL) [43]. The IC_50_ values of Greek pollen [4] and pollen from Portugal and New Zealand [44] were 0.181 ± 1.70 mg/mL and 0.04–0.5 mg/mL, respectively, which are within the range of our Algerian bee pollen samples from several regions. When comparing the FRAP assay results with those of other researchers, we observe notable similarities and differences, providing valuable insights into antioxidant capacity variations. Studies evaluating the antioxidant capacity of bee pollen extracts from various floral regions in Morocco [38] reported EC_50_ values ranging from 0.133 ± 0.036 to 0.790 ± 0.175 mg/mL. Similarly, analyses of ethanolic pollen extracts from apiaries in Bahia, Brazil [41] showed antioxidant activity values between 1.45 ± 0.03 and 8.77 ± 0.23 mg/mL. These findings suggest that the antioxidant activity of Moroccan and Brazilian bee pollen is generally lower than that of Algerian samples. The antioxidant capacity of bee pollen extracts has been extensively evaluated using the FRAP method, though results often differ in their expression. Alimoglu et al. [5] investigated 11 bee pollen samples from Turkey, reporting FRAP values between 3.42 ± 0.07 mg Trolox/g and 16.03 ± 0.25 mg Trolox/g. Similarly, Ulusoy and Kolayli [45] analyzed 13 Anzer bee pollen samples, and Karkar et al. [46], examined 10 samples from Turkey, finding FRAP values ranging from 11.77 ± 0.63 mmol Trolox/g to 4.75 ± 0.02 mmol Trolox/g and from 1.53 ± 0.06 mg Trolox/g to 4.75 ± 0.02 mg Trolox/g, respectively. Additionally, coriander bee pollen extracts demonstrated a wide range of FRAP values, from 32.54 ± 0.57 to 103.98 ± 0.82 mmol Fe^2^⁺/g, with the highest values [7]. These variations highlight the diverse antioxidant potential of bee pollen across different geographical and botanical origins. Limited data exists on total antioxidant capacity using the phosphomolybdenum method. In this study, pollen samples exhibited lower TAC compared to Moroccan samples (3.98 ± 0.16 mg EAA/g to 9.69 ± 0.34 mg EAA/g) [38] and Algerian samples (808.2–3311 mg/100 g) [40], but higher values than those reported by Rebiai and Lanez [47]. Significant variations in TPC, TFC, and antioxidant capacities of bee pollen have been observed worldwide. These differences are influenced by factors such as the choice of standards (e.g., gallic acid, pinocembrin, or chlorogenic acid), the extraction solvent (e.g., methanol, ethanol, or dichloromethane), and the extraction method. Additionally, the botanical and geographical origins of bee pollen, along with environmental factors like soil type, climate conditions, and beekeeping practices, play a crucial role in determining its composition [30,38,46].

Bee pollen has garnered increased attention for its phenolic profile, which has been fairly well reported in the literature [48]. In this study, the phenolic compounds identified in Algerian bee pollen samples align with the findings of Bayram et al. [49], who reported rutin as the dominant compound in Turkish bee pollen. Additionally, resveratrol and kaempferol were present in considerable amounts, whereas coumaric acid was detected in lower concentrations and was absent in samples P1, P2, P3, P7, P14, and P15. However, research on Algerian bee pollen remains scarce, highlighting the need for further studies. Hemmami et al. [40], analyzed 13 Algerian bee pollen, identifying high levels of rutin (6751.996 ± 168.799 µg/g), quercetin (20,749.05 ± 518.726 µg/g), and gallic acid (648.558 ± 12.971 µg/g). All 13 Hemmami and coworker’s samples contained gallic acid, p-coumaric acid, and quercetin. However, different from our results specific compounds, i.e., vanillin, chlorogenic acid, naringin, vanillic acid, rutin, and caffeic acid, were completely lacking in several samples, and only one sample contained all nine studied phenolic compounds. Aylanc et al. [26] detected caffeic acid and p-coumaric acid only in one Moroccan sample, with concentrations of 150 µg/g and 200 µg/g, respectively. Similarly, Kahraman et al. [30] found kaempferol (3264.5 µg/g) in Turkish bee pollen but did not identify epicatechin, syringic acid, vanillic acid, or catechin. For instance, Karkar et al. [46] did not detect gallic acid in ethanolic extracts of Turkish chestnut bee pollen but found low levels of syringic acid, kaempferol, and myricetin. Alimoglu et al. [5] reported different profiles using ethyl acetate and dichloromethane extracts, demonstrating that solvent selection significantly affects phenolic extraction. Thus, bee pollen extracts contain phenolic compounds, whose profiles differ markedly, as clearly documented in the literature.

Numerous studies have explored the relationship between phenolic content and the antioxidant capacity of bee pollen [3,50,51], and recent findings highlight a strong positive association [26,28,29,47]. Limmongkon et al. [52] reported a significant correlation (r = 0.773) between the DPPH and FRAP assays, both of which measure the electron transfer potential of antioxidants. Our results are consistent with this observation, further supporting the link between phenolic compounds and their antioxidant efficacy.

Certain bee pollen samples present higher TPC values and lower DPPH IC_50_. The presence of specific phenolic compounds with high radical-scavenging efficiency may explain this relationship. This trend is expected, as phenolic compounds are known for their ability to scavenge free radicals [53]. The strong correlations may be due to the presence of flavonols such as rutin, kaempferol, and myricetin derivatives. Their planar structures, influenced by hydroxyl groups at position 3, facilitate radical capture through conjugation and electron delocalization. Moreover, the high number of hydroxyl groups contributes significantly to their potency as electron scavengers. Despite this, several studies have concluded that antioxidant capacity is not necessarily linked to total phenolic content [46,54,55,56]. In this study, the sample with the highest TPC (P2) showed the lowest TAC. This indicates that the presence of specific phenolic compounds, rather than their concentration, may play a more critical role. Synergistic and antagonistic effects among phenolic compounds and interactions with other phytochemicals in the extract further influence antioxidant activity [52]. Environmental factors, such as soil conditions and plant physiology, also impact free radical reactions and the antioxidant capacity of bee pollen [34,41]. Furthermore, the collection period significantly affects antioxidant activity, with higher values observed during UV-intense periods, particularly from early to late summer [3]. Consequently, proper management practices and the timing of collection are crucial for optimizing the chemical and functional properties of bee pollen.

While this study provides valuable insights into the botanical origin, phytochemical profile, and antioxidant activity of bee pollen from the Mila region, certain limitations should be acknowledged. The sample size, though representative, could be expanded to improve the generalizability of the findings. Additionally, while three antioxidant assays (DPPH, FRAP, and TAC) were employed, incorporating a broader range of assays would provide a more comprehensive evaluation of the antioxidant potential. Further, the HPLC analysis, which identified key phenolic compounds, could be extended to include a wider range of bioactive compounds for a more detailed phytochemical characterization. Despite these limitations, the findings of this study contribute to the growing body of knowledge on natural antioxidants and highlight the need for further investigations to overcome these challenges.

## 5. Conclusions

This study investigated bee pollen samples from the Mila region in northeastern Algeria. Bee pollen is recognized as a valuable functional food due to its high levels of phenolics and strong antioxidant properties. These compounds are known to offer various health benefits. The study aimed to determine the botanical origin of the pollen samples, analyze their antioxidant capacity using different methods (DPPH, FRAP, and TAC), and characterize their phenolic composition using HPLC. The results showed significant variations in phenolic content and antioxidant activity among pollen samples from different plant sources. Phenolic compounds were identified as the primary contributors to the antioxidant properties of the pollen. HPLC analysis revealed the presence of six phenolic compounds across all samples, with rutin, myricetin, and naringenin being the most abundant. Moreover, the results suggest that bee pollen from the Mila region could contribute to human health as a powerful natural antioxidant and should be applied to various fields of food and pharmaceutical industries. However, the study emphasizes the need and opportunity for further research, increasing such as the number of samples analyzed, expanding the research to other regions of Algeria, and exploring other research approaches to gain a deeper understanding of the therapeutic and economic value of Algerian bee pollen.

## Figures and Tables

**Table 1:** Geographical location and harvesting period of bee pollen samples.

Sample	Date of Harvest	Geographical Origin	Latitude (°N)	Longitude (°E)	Altitude (m)
P1	Spring 2023	Tessala Lamtai	36.4500	6.2667	600
P2	Spring 2023	Tassadane Haddada	36.3667	6.2500	700
P3	Spring 2023	Grarem Gouga	36.4000	6.5667	500
P4	Spring 2023	Derradji bousselah	36.3833	6.3333	550
P5	Spring 2023	Rouached	36.4667	6.1167	650
P6	Spring 2023	Zeghaia	36.4500	6.4000	450
P7	Spring 2023	Hamala	36.3667	6.4167	600
P8	Spring 2023	Rouached	36.4667	6.1167	650
P9	Spring 2023	Minar Zarza	36.3167	6.2000	750
P10	Summer 2023	Amira Arres	36.3000	6.2833	800
P11	Spring 2023	Tessala Lamtai	36.4500	6.2667	600
P12	Spring 2023	Oued Althmenia	36.4667	6.3667	450
P13	Spring 2023	Tassala Lemtai	36.4500	6.2667	600
P14	Spring 2023	Mila	36.4500	6.2667	710
P15	Spring 2023	Tassadane Haddada	36.3667	6.2500	700

**Table 2:** Retention times (Rt), detection, and quantification the phenolic standards.

Time (min)	RT (min)	LOD (µg/mL)	LOQ (µg/mL)
Gallic Acid	7.33	0.03	0.09
Hidroxycinnamic acid	17.00	0.03	0.09
Chlorogenic acid	21.33	0.02	0.06
Catechin	22.94	0.04	0.13
Vanillic acid	25.08	0.01	0.04
Caffeic acid	27.25	0.02	0.06
Syringic acid	28.00	0.03	0.10
Epicatechin	33.00	0.04	0.13
Trans cinnamic acid	38.35	0.11	0.33
p-Coumaric acid	44.74	0.04	0.12
Ferulic acid	53.66	0.09	0.28
Rutin	65.61	0.08	0.24
Myricetin	71.64	0.02	0.06
Resveratrol	74.55	0.04	0.13
Quercetin	79.10	0.01	0.04
Naringenin	84.95	0.05	0.16
Kaempferol	86.50	0.04	0.12

**Table 3:** Palynological analysis of bee pollen samples.

Sample	Predominant Pollen Grains (>45%)	Secondary Pollen Grains (16–45%)	Important Minor Pollen (3–15%)	Minor Pollen (<3%)	Classification
P1	*Cistus* type ^a^ (63%)	*Brassica* type ^b^ (16%)	*Trifolium repens* ^j^ (11%), *Onobruchis* sp. ^j^ (7%)	*Anthemis* type ^e^, *Castanea sativa* ^i^, *Centaurea* sp. ^e^, Fabaceae ^j^, Rosaceae ^k^	Monofloral
P2	*Cistus* type ^a^ (82%)	/	*Olea europaea* ^l^ (14%)	*Quercus* sp. ^i^	Monofloral
P3	*Cistus* type ^a^ (65%)	*Erica* sp. ^c^ (25%)	*Quercus* sp. ^i^ (7%)	*Oxalis* sp. ^m^	Monofloral
P4	*Brassica* type ^b^ (82%)	/	*Meliotus* type ^j^ (15%)	Rosaceae ^k^, *Prunus* sp. ^k^, *Cistus* type ^a^, *Carduus* type ^e^, Apiaceae ^d^	Monofloral
P5	/	*Brassica* type ^b^ (39%), *vitex* sp. ^g^ (41%)	*Melilotus* type ^j^ (10%), *Erica* sp. ^c^ (7%)	*Cistus* type ^a^	Polyfloral
P6	*Brassica* type ^b^ (50%)	*Erica* sp. ^c^ (36%)	*Rubus* sp. ^k^ (6%), *Aster* type ^e^ (4%), *Taraxacum* type ^e^ (4%)	*Capparis spinosa* ^n^, *Cistus* type ^a^	Monofloral
P7	*Brassica* type ^b^ (55%)	*Cistus* type ^a^ (29%),	*Melilotus* type ^j^ (8%), *Lotus* sp. ^j^ ( 5%)	*Rubus* sp. ^k^, *Anthemis* type ^e^	Monofloral
P8	/	*Brassica* type ^b^ (43%), *vitex* sp. ^g^ (42%)	*Melilotus* type ^j^ (7%), *Erica* sp. ^c^ (5%)	*Cistus* type ^a^	Polyfloral
P9	*Pimpinella anisum* ^d^ (49%)	/	*Hedysarum coronarium*^j^ (10%), *Taraxacum* type ^e^ (15%), *Anthemis* type ^e^ (12%), *Quercus* sp. ^i^ (11%)	*Rubus* sp. ^k^, *Eucalyptus* sp., *Carduus* type ^e^, *Trifolium* type ^j^, *Cistus* type ^a^	Monofloral
P10	*Erica* sp. ^c^ (53%)	*Melilotus* sp. ^j^ (32%)	Asreraceae ^e^ (7%), *Brassica* type ^b^ (5%),	Apiaceae ^d^, Rosaceae ^k^, *Carduus* type ^e^, *Asparagus* type ^p^, *Alluim* sp. ^h^, *Rubus* sp. ^k^	Monofloral
P11	*Cistus* type ^a^ (47%)	Liliaceae (44%)	*Carduus* type ^e^ (9%)	/	Monofloral
P12	*Brassica* type ^b^ (71%)	Apiaceae ^d^ (26%)	/	*Carduus* type ^e^	Monofloral
P13	*Aster* type ^e^ (52%)	*Cistus* type ^a^ (23%)	*Erica* sp. ^c^ (12%), *Quercus* sp. ^i^ (10%)	*Asparagus* type ^p^, *Anthemis* type ^e^, *Taraxacum* type ^e^, Fabaceae ^j^	Monofloral
P14	/	Oleaceae ^l^ (39%), *Tamarix* sp. ^f^ (29%)	*Quercus* sp. ^i^ (8%), Lamiaceae ^g^ (9%), *Brassica* type ^b^ (5%), *Centaurea* sp. ^e^ (7%)	*Carduus* type ^e^, *Taraxacum* type ^e^, *Plantago* sp. ^q^, *Trifolium pratanse* ^j^, *Eucalyptus* sp. ^o^, *Genista* type ^j^, *Melilotus* sp. ^j^	Polyfloral
P15	/	*Artemesia* sp. ^e^ (37%), *Brassica* type ^b^ (21%), *Helianthus annuus* ^e^ (41%)	*Alluim* sp. ^h^ (4%)	*Trifolium repens* ^j^, *Cistus* type ^a^, Rosaceae ^k^, *Tamarix* sp. ^f^, Chenopodiaceae, Apiaceae ^d^	Polyfloral

Different letters (a–q) indicate the families: ^a^ Cistaceae, ^b^ Brassicaceae, ^c^ Ericaceae, ^d^ Apiaceae, ^e^ Asteraceae, ^f^ Tamaricaceae, ^g^ Lamiaceae, ^h^ Alliaceae, ^i^ Fagaceae, ^j^ Fabaceae, ^k^ Rosaceae, ^l^ Oleaceae, ^m^ Oxalidaceae, ^n^ Capparaceae, ^o^ Myrtaceae, ^p^ Asparagaceae, and ^q^ Plantaginaceae.

**Table 4:** Antioxidant content and activities of the ethanolic extract of bee pollen.

Sample	Total Phenolic Content (TPC) (mgGAE/g)	Total Flavonoid Content (TFC) (mg QE/g)	DPPH Radical Scavenging Assay (IC_50_) (mg/mL)	Total Antioxidant Capacity (TAC) (EAA mg/100 g)	Reducing Power (FRAP) (EC _50_) (mg/mL)
P1	12.37 ± 0.19 ^b,c^	2.80 ± 0.33 ^c,d^	0.26 ± 0.01 ^a,b^	401.52 ± 1.13 ^d,e^	0.12 ± 0.02 ^c,d^
P2	23.49 ± 1.48 ^i^	2.99 ± 0.02 ^d^	0.34 ± 0.04 ^a,b,c^	465.56 ± 25.63 ^g^	0.09 ± 0.00 ^a,b^
P3	19.69 ± 0.69 ^i^	2.95 ± 0.07 ^d^	0.52 ± 0.01 ^d,e,f^	262.17 ± 3.41 ^a^	0.06 ± 0.00 ^a^
P4	19.05 ± 1.90 ^h,i^	1.70 ± 0.05 ^a,b^	0.25 ± 0.03 ^a,b^	612.54 ± 5.12 ^i^	0.09 ± 0.00 ^a,b^
P5	15.91 ± 0.64 ^e,f,g^	4.74 ± 0.24 ^f,g^	0.67 ± 0.05 ^f^	672.36 ± 3.41 ^j^	0.10 ± 0.00 ^b,c^
P6	13.69 ± 0.50 ^c,d,e^	2.43 ± 0.05 ^c,d^	0.42 ± 0.00 ^b,c,d,e^	246.56 ± 10.25 ^a^	0.13 ± 0.00 ^c,d^
P7	17.09 ± 0.32 ^g,h^	3.92 ± 0.24 ^e^	0.50 ± 0.04 ^b,c,d,e,f^	595.96 ± 6.83 ^i^	0.12 ± 0.00 ^a,b^
P8	17.18 ± 0.27 ^f,g^	1.77 ± 0.21 ^a,b^	0.38 ± 0.01 ^a,b,c^	373.26 ± 5.12 ^c,d^	0.13 ± 0.01 ^c,d^
P9	14.70 ± 0.37 ^c,d,e,f^	5.57 ± 0.27 ^h^	0.53 ± 0.00 ^d,e,f^	551.92 ± 1.13 ^h^	0.12 ± 0.00 ^b,c,d^
P10	14.87 ± 1.02 ^d,e,f^	4.28 ± 0.31 ^e,f^	0.58 ± 0.05 ^e,f^	592.03 ± 8.54 ^i^	0.13 ± 0.01 ^d^
P11	8.28 ± 0.67 ^a^	2.94 ± 0.10 ^d^	1.12 ± 0.15 ^g^	354.46 ± 6.83 ^b,c^	0.29 ± 0.00 ^f^
P12	13.42 ± 0.30 ^c,d^	3.74 ± 0.33 ^e^	0.21 ± 0.00 ^a^	677.14 ± 12.81 ^j^	0.07 ± 0.00 ^a^
P13	7.72 ± 0.29 ^a^	1.48 ± 0.00 ^a^	0.67 ± 0.07 ^f^	421.12 ± 8.54 ^e,f^	0.20 ± 0.00 ^e^
P14	11.05 ± 0.08 ^b^	4.98 ± 0.09 ^g,h^	0.63 ± 0.01 ^f^	433.08 ± 17.09 ^f^	0.15 ± 0.01 ^d^
P15	8.52 ± 0.08 ^a^	2.28 ± 0.03 ^b,c^	0.99 ± 0.08 ^g^	330.19 ± 5.98 ^b^	0.18 ± 0.01 ^e^
Average	14.32	3.26	0.54	476.24	0.12

We compared differences between the resulting TPC, TFC, DPPH, TAC, and FRAP of all BPEs using one-way ANOVA and Tukey’s multiple comparisons tests. Within the same column, different letters (a–j) indicate significant differences (*p* < 0.05).

**Table 5:** HPLC analysis of phenolic compounds of bee pollens (µg/g).

Sample	p-Coumaric Acid	Rutin	Myricetin	Resveratrol	Naringenin	Kaempferol
P1	Not detected	124.8	995.4	186.2	177.1	37.0
P2	Not detected	103.8	532.1	59.6	84.5	39.5
P3	Not detected	488.1	2081.7	377.7	7.6	62.0
P4	89.0	48.1	255.1	291.2	161.0	347.6
P5	77.6	38.3	36.1	453.4	488.7	11.5
P6	40.9	613.1	89.9	255.2	112.1	5.4
P7	Not detected	187.0	977.2	69.7	102.1	4.6
P8	23.2	12.9	56.7	160.6	625.9	90.8
P9	32.8	1052.7	2062.1	48.4	1418.1	172.4
P10	64.8	23.8	52.8	156.3	220.8	21.8
P11	Not detected	32.2	10.5	8.4	705.9	6.6
P12	130.3	496.3	883.4	413.1	205.6	67.0
P13	3.9	71.1	163.2	122.7	192.5	17.2
P14	Not detected	361.2	696.2	149.3	193.3	22.7
P15	Not detected	11.4	26.7	149.2	97.8	13.7
Average	31.3	303.3	700.1	191.5	354.6	53.5

## Data Availability

Data is contained within the article.
